# Laryngeal Burn from a Sweet Potato: A Case Report

**DOI:** 10.51894/001c.11641

**Published:** 2020-01-30

**Authors:** Steven Pinther, Juliana Codino, Adam Rubin

**Affiliations:** 1 Otolaryngology SCS/MSUCOM; 2 Speach and Language Pathology Lakeshore Professional Voice Center; 3 Otolaryngology Lakeshore Professional Voice Center

**Keywords:** microwaved foods, oral burn, laryngeal burn, dysphagia

## Abstract

**CONTEXT:**

Thermal injury to the larynx and other pharyngeal structures as a result of food ingestion is a rare occurrence, particularly in an adult population. Since the 1970’s, only a few cases have been reported in the literature.

**CASE PRESENTATION:**

We present the case of a male in their early 30’s with a history of left sided spastic hemiparesis and unilateral vocal fold paresis who ingested a sweet potato which resulted in supraglottic burns. The patient denied any prior swallowing difficulties. Conservative therapy with steroids, proton pump inhibitors (PPI’s) and antibiotics were sufficient for full recovery without any lasting sequelae.

**CONCLUSIONS:**

This case demonstrates how careful attention should be paid to food temperature particularly in patients at higher risk of dysphagia. It also demonstrates how prompt diagnosis and implementation of appropriate medications can prevent permanent and debilitating damage.

## INTRODUCTION

Thermal injury to the larynx and other pharyngeal structures, such as the tongue, airway, or esophagus, can result from ingestion of excessively hot foods. Thermal injury from the ingestion of hot food is a rare occurrence, particularly in an adult population.^1–3^ Since the 1970’s, only a few cases have been reported in the literature.^4^ This case demonstrates how careful attention should be paid to food temperature particularly in patients at higher risk of dysphagia. These patients may include those with neurologic conditions such as multiple sclerosis, Parkinson’s disease, amyotrophic lateral sclerosis (ALS), stroke patients, diabetes and those with other neurodegenerative diseases. These diseases affect the capacity of the nerve to transmit signals in a normal speed and fashion, which in turn can lead to decreased sensation and reaction times.^5^ In particular with regards to feeding, if compromised individuals are not careful they may introduce dangerously hot substances into their mouths and be unable to reflexively spit them out which could result in thermal injury to oral, airway, and other gastrointestinal structures if the food bolus is swallowed before cooling. This can also be seen in elderly patients who have “normal” nerve degeneration with age. This can lead to diminished throat sensation and gag reflexes becoming absent or reduced.

## CASE REPORT

We present the case of a male in their early 30’s with a history of left sided spastic hemiparesis, a condition leading to muscle spasms on one side of the body as a result of neurologic injury sustained in a motor vehicle accident 11 years prior. As a result of the accident this individual was unable to move his limbs normally due to muscle contractions, and he also had unilateral vocal fold paresis, a condition in which the vocal cord movement is restricted but not completely paralyzed. However, he denied any prior history of problems or difficulty with swallowing prior to this event. This individual ingested a sweet potato which resulted in supraglottic (anatomic region above the true vocal cords) burns. Conservative therapy with steroids, proton pump inhibitors (PPIs) and antibiotics were sufficient for full recovery without any lasting sequelae.

The patient presented to the emergency department (ED) 8 hours after ingesting a sweet potato that was heated in the microwave. According to the patient, the potato did not feel excessively hot to the touch, until he put it in his mouth and bit into it. He attempted to swallow it, but it became lodged in his throat causing extreme discomfort. He was able to expectorate the bolus after a few seconds along with some blood tinged sputum. His pain became progressively worse. He began to note a change in voice quality and increased difficulty breathing. In the ED, the patient was treated with 10 mg of IV Dexamethasone and “Kool’s solution” which consisted of diphenhydramine, Lidocaine, and Maalox. A lateral neck X-ray was performed which suggested thickening of the epiglottis and aryepiglottic folds (AE folds), anatomic structures located in the back of the mouth where the transition from oral cavity to the airway and the esophagus occurs. ENT consult was placed, and flexible laryngoscopy revealed moderate areas of denudation (where normal overlying tissue had been lost or removed) by thermal injury, as well as swelling of the epiglottis, AE folds, and bilateral arytenoid cartilages (small structures that make up the back third of the vocal cords and help protect the airway).^6^ The patient was admitted for airway observation and treated with 3 more doses of steroids, antibiotics and proton-pump inhibitor. His hospital course was uneventful and he was discharged after 18 hours of observation with Augmentin, Nystatin, PPI, and a cold liquid diet. He was referred for a 1-week follow up appointment with laryngology.

**Figure 1:**
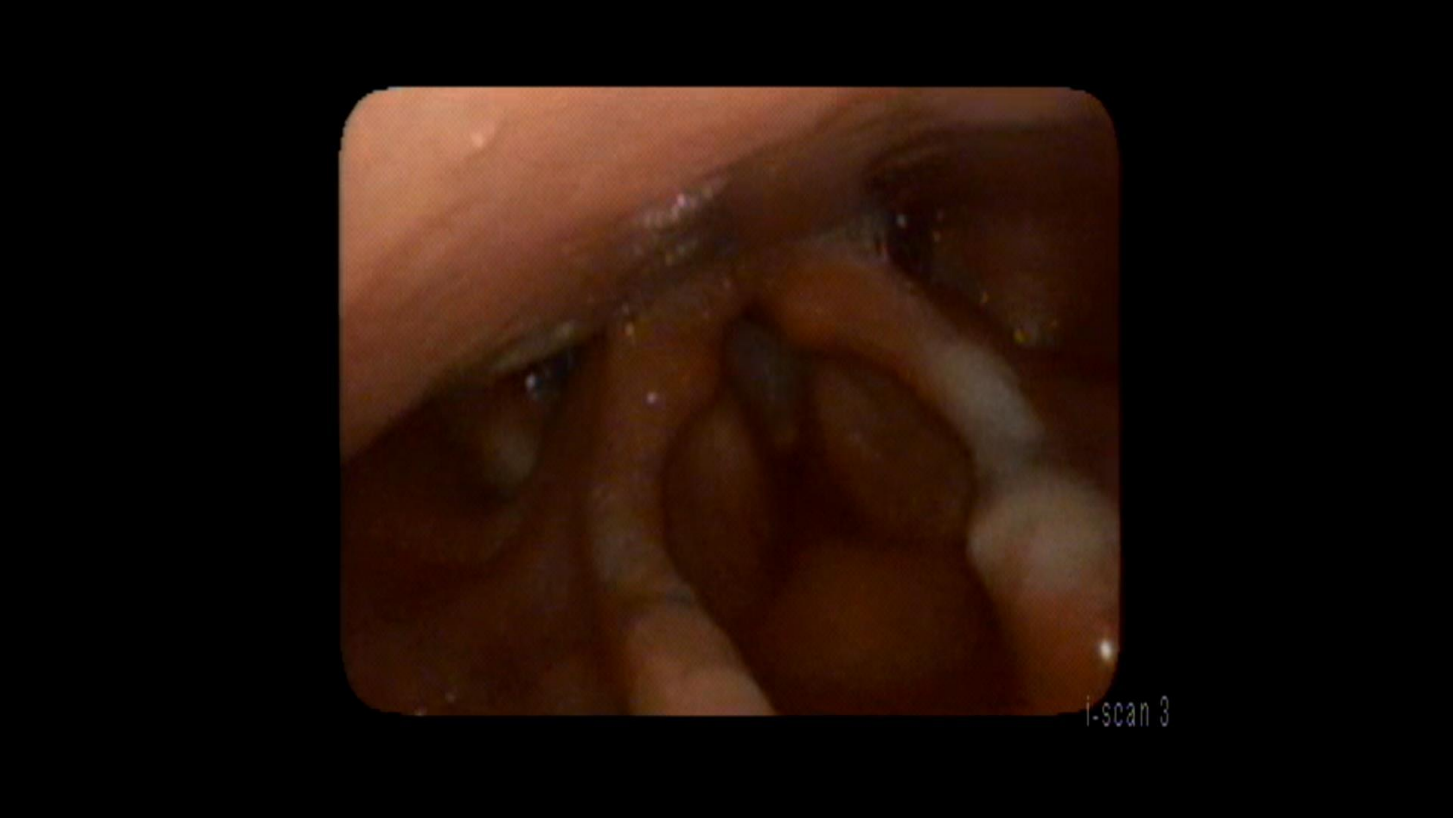
Approx 1 week after injury. The white appearing lesions noted on the edge of the epiglottis extending down AE folds to the arytenoids on both sides with the patients left side being worse than the right.

Upon follow up, videostroboscopy (a high magnification, slow motion video recording that allows for visualization of vibratory capability of the true vocal cords (TVF’s)) was performed. His burns were found to be healing, but there were still areas of denudation and granulation along the aryepiglottic folds (Figure 1).

**Figure 2:**
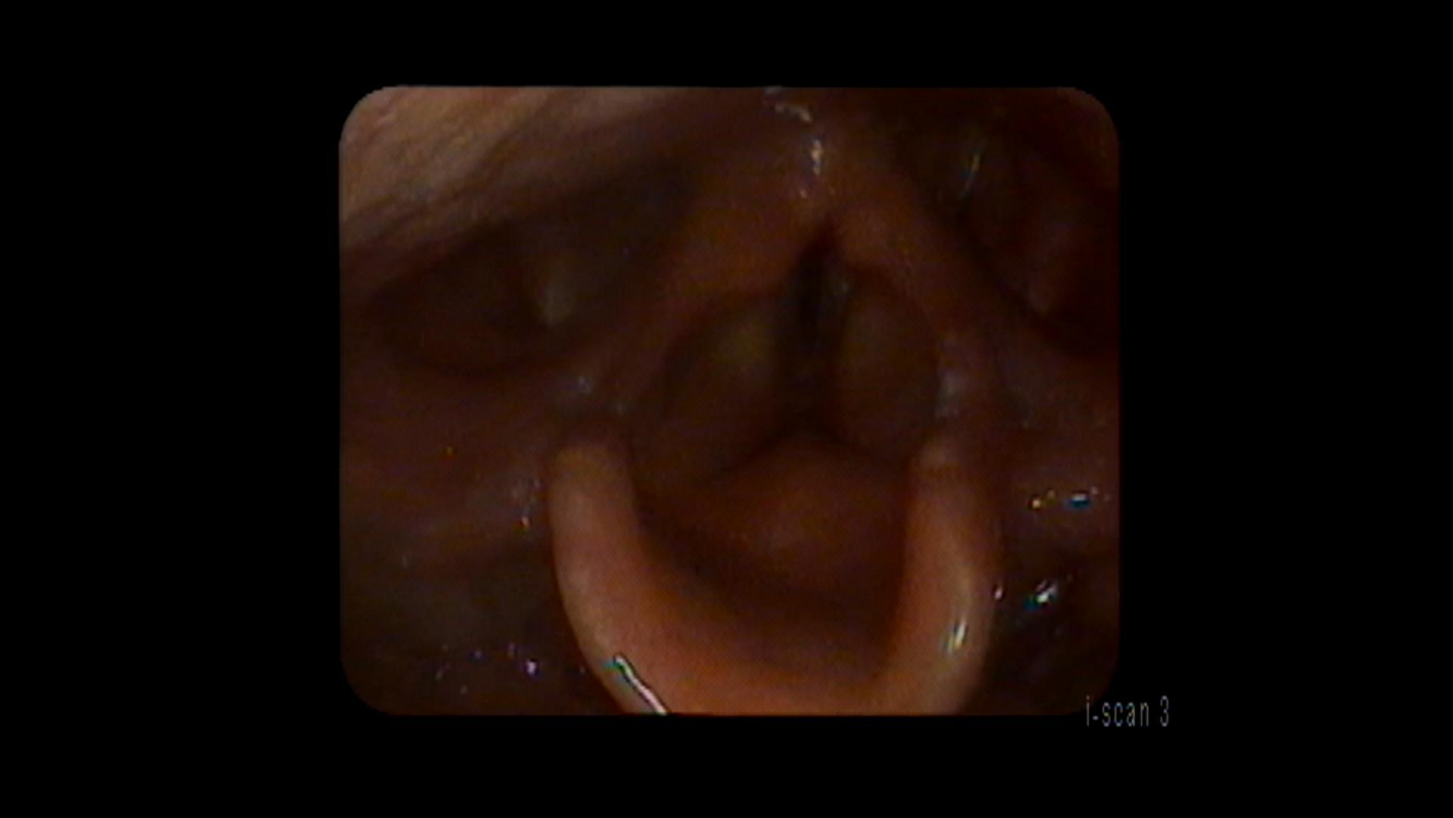
Approx. 3 wks after injury. This shows near complete resolution of previously denuded areas and near complete resolution of the previously noted edema.

There was also impaired vibration of the true vocal folds. He reported that his voice was improving and the pain was nearly resolved. His diet was advanced to regular foods. He was seen back two weeks later and reported that his pain had completely resolved. His voice was back to normal and he was singing again. Videostroboscopy revealed nearly complete resolution of the granulation tissue (Figure 2). His TVF vibration had returned to normal. He had no long lasting sequelae from this injury and returned to his normal baseline function.

## DISCUSSION

When dealing with microwaved foods it is important to realize that the external temperature may not be reflective of the deeper layers of the food. There can be large fluctuations in food temperature based on microwave power and the type of foods being heated.^1,2^ Excessively hot food bolus temperature can lead to significant oral, pharyngeal, laryngeal or esophageal injury.^7,8^ Particular attention should be paid to patients with neurologic disease, injury, or other conditions as outlined above, that would predispose them to oropharyngeal dysphagia.^5^ While at risk patients may be able to function at a high level the implications of them having decreased reflexes or reaction times can lead to an increased propensity for and severity of injury.^9^ Sequelae from these injuries, if severe enough, can lead to scarring of the esophagus, larynx, and other oral structures. This could further lead to complications such as feeding tube dependence, tracheostomy tube dependence, or need for multiple surgeries to address soft tissue bands causing strictures and narrowing of structures such as the esophagus and glottis.^10^ These scar bands can form anywhere that thermal injury penetrates deep to the protective mucosal layers lining the oral, laryngeal, and intestinal structures.

While this patient denied any baseline dysphagia, he likely had some subclinical dysphagia due to his neurologic deficits.^11^ Stasis of the bolus in any part of the digestive tract will increase risk of thermal injury. Dysphagic and other at-risk patients, family members, and caretakers should be educated as to the risks of thermal injury from extremes in bolus temperature. They should take precautions to avoid excessive heating of foods in microwaves and always ensure that food is adequately cooled throughout to prevent thermal injury.

With regards to intervention and whether or not patients should be referred for further evaluation after a thermal injury, it is the authors opinion that any individual who has voice changes, or difficulty with breathing after a thermal injury, should seek immediate care at the nearest emergency department as these ^5^are signs of airway swelling and possible impending airway emergency. For those individuals who have pain and difficulty with swallowing for more than 48 hrs after a thermal injury, they should seek evaluation by their primary provider who may choose to manage medically or may choose to refer to a specialist for further evaluation and management.

## CONCLUSIONS

In conclusion the authors suggest that caretakers and those helping to care for those at risk of dysphagia and laryngeal burns be given proper education and precautions to avoid the potential of thermal injury of the oral cavity and other gastrointestinal and airway structures. Further instructional materials for providers, caregivers, and patients should be created to assist in the educational process of identifying at risk individuals and helping to prevent thermal injury from extreme food bolus temperatures.^5^

### Conflict of Interest

The authors declare no conflict of interest.

## References

[ref-11424] Goldberg Richard M., Lee Stanford, Line Warren S. Jr. (1990). Laryngeal burns secondary to the ingestion of microwave-heated food. The Journal of Emergency Medicine.

[ref-11425] Iyama Keita, Ueki Tomohiro, Yamano Shuhei, Tajima Goro, Inokuma Takamitsu, Hirao Tomohito, Yamashita Kazunori, Nagatani Atsuko, Tasaki Osamu (2016). An adult case of laryngopharyngeal burn by drinking hot water. Acute Medicine & Surgery.

[ref-11426] Wu Chia-Hsien, Bair Ming-Jong, Lin I-Tsung, Lee Yuan-Kai, Chen Huan-Lin (2015). Early endoscopic finding of esophageal thermal injury after having spicy hot pot. Advances in Digestive Medicine.

[ref-11427] Lieberman DAVID A. (1982). Esophageal burn and the microwave oven. Annals of Internal Medicine.

[ref-11428] Wang C.M., Tsai T.T., Wang S.H., Wu Y.R. (2019). Does the M.D. Anderson Dysphagia Inventory correlate with dysphagia-limit and the Unified Parkinson Disease Rating Scale in early-stage Parkinson's disease?. J Formos Med Assoc.

[ref-11429] https://teachmeanatomy.info/neck/viscera/larynx/.

[ref-11430] Hyo Yukiyoshi, Fukutsuji Kenji, Fukushima Hisaki, Harada Tamotsu (2017). Two cases of thermal burns of the larynx in older men. Auris Nasus Larynx.

[ref-11431] Goto R., Miyabe K., Mori N. (2002). Thermal burn of the pharynx and larynx after swallowing hot milk. Auris Nasus Larynx.

[ref-11434] Pelicioni Paulo H.S., Pereira Marcelo P., Lahr Juliana, Rodrigues Mariana M.L., de Morais Luana C., Moraes Renato, Gobbi Lilian T.B. (2019). Motor adjustments during time-constrained sit-to-walk in people with Parkinson's disease. Experimental Gerontology.

[ref-11432] Reid A., Ha J.F. (2019). Inhalational injury and the larynx: A review. Burns.

[ref-11433] Briani C., Marcon M., Ermani M.. (1998). Radiologcal evidence of subclinical dysphagia in motor neuron disease. J Neurol.

